# *Ex vivo* mammalian prions are formed of paired double helical prion protein fibrils

**DOI:** 10.1098/rsob.160035

**Published:** 2016-05-04

**Authors:** Cassandra Terry, Adam Wenborn, Nathalie Gros, Jessica Sells, Susan Joiner, Laszlo L. P. Hosszu, M. Howard Tattum, Silvia Panico, Daniel K. Clare, John Collinge, Helen R. Saibil, Jonathan D. F. Wadsworth

**Affiliations:** 1 MRC Prion Unit and Department of Neurodegenerative Disease, UCL Institute of Neurology, National Hospital for Neurology and Neurosurgery, Queen Square, London WC1N 3BG, UK; 2 Institute of Structural and Molecular Biology, Department of Biological Sciences, Birkbeck College, University of London, Malet Street, London WC1E 7HX, UK

**Keywords:** prion, prion disease, prion protein, prion structure, electron tomography

## Abstract

Mammalian prions are hypothesized to be fibrillar or amyloid forms of prion protein (PrP), but structures observed to date have not been definitively correlated with infectivity and the three-dimensional structure of infectious prions has remained obscure. Recently, we developed novel methods to obtain exceptionally pure preparations of prions from mouse brain and showed that pathogenic PrP in these high-titre preparations is assembled into rod-like assemblies. Here, we have used precise cell culture-based prion infectivity assays to define the physical relationship between the PrP rods and prion infectivity and have used electron tomography to define their architecture. We show that infectious PrP rods isolated from multiple prion strains have a common hierarchical assembly comprising twisted pairs of short fibres with repeating substructure. The architecture of the PrP rods provides a new structural basis for understanding prion infectivity and can explain the inability to systematically generate high-titre synthetic prions from recombinant PrP.

## 1. Introduction

Prions are unique pathogens, devoid of significant coding nucleic acid, which cause lethal neurodegenerative diseases in mammals, including scrapie in sheep and goats, bovine spongiform encephalopathy (BSE) in cattle and Creutzfeldt–Jakob disease (CJD) in humans [1–3]. They are hypothesized to be fibrillar or amyloid forms of prion protein (PrP), which self-propagate by means of seeded protein polymerization [1,2]. It is increasingly recognized that similar seeding processes may be involved in Alzheimer's disease and other degenerative conditions [4].

To date the three-dimensional structure of infectious prions and how this differs from non-infectious amyloid fibrils remains unknown. Prion-infected brain contains infectious PrP assemblies of variable size that can be fractionated [5–9], but the production of highly homogeneous material of demonstrable high specific infectivity to allow direct correlation of particle structure with infectivity has proved extremely challenging. Moreover, the knowledge that partially purified prions can be fragmented *in vitro* to generate smaller oligomeric PrP assemblies of high specific prion infectivity [6] has not yet facilitated the provision of material suitable for high-resolution structural studies. Recently, however, we developed new methods [10] to obtain exceptionally pure preparations of intact prions from mouse brain, and showed that pathogenic PrP in these preparations is assembled into rod-like assemblies (PrP rods) akin to those described by Prusiner and colleagues [1,11]. Importantly, the PrP rods that we prepare contain disease-related PrP at greater than 99% protein purity [10], and these are therefore devoid of detectable protein contaminants which might confound structural studies. The fact that these preparations have very high titres of infectious prions which faithfully transmit prion strain-specific phenotypes when inoculated into mice [10] makes them eminently suitable for detailed study. Here, we have used the precision of cell culture prion infectivity assays [10,12–16] to define the physical relationship between the PrP rods and prion infectivity and have used electron tomography to define their architecture.

We show that PrP rods isolated *ex vivo* from multiple prion strains are intrinsically infectious. Furthermore, we show that the PrP rods are formed in infected brain and are not, as previously thought, an artefact of extraction protocols involving proteases and detergents [17]. They have a common hierarchical assembly comprising twisted pairs of short fibres with repeating substructure. Such paired fibre assembly is markedly different to the long single fibre organization of non-infectious PrP fibrils generated from recombinant PrP. This novel architecture now provides a new basis for understanding the distinctive properties of prions compared to non-infectious amyloid structures.

## 2. Results

### 2.1. Prion protein rods are intrinsically associated with prion infectivity

**Figure 1.**
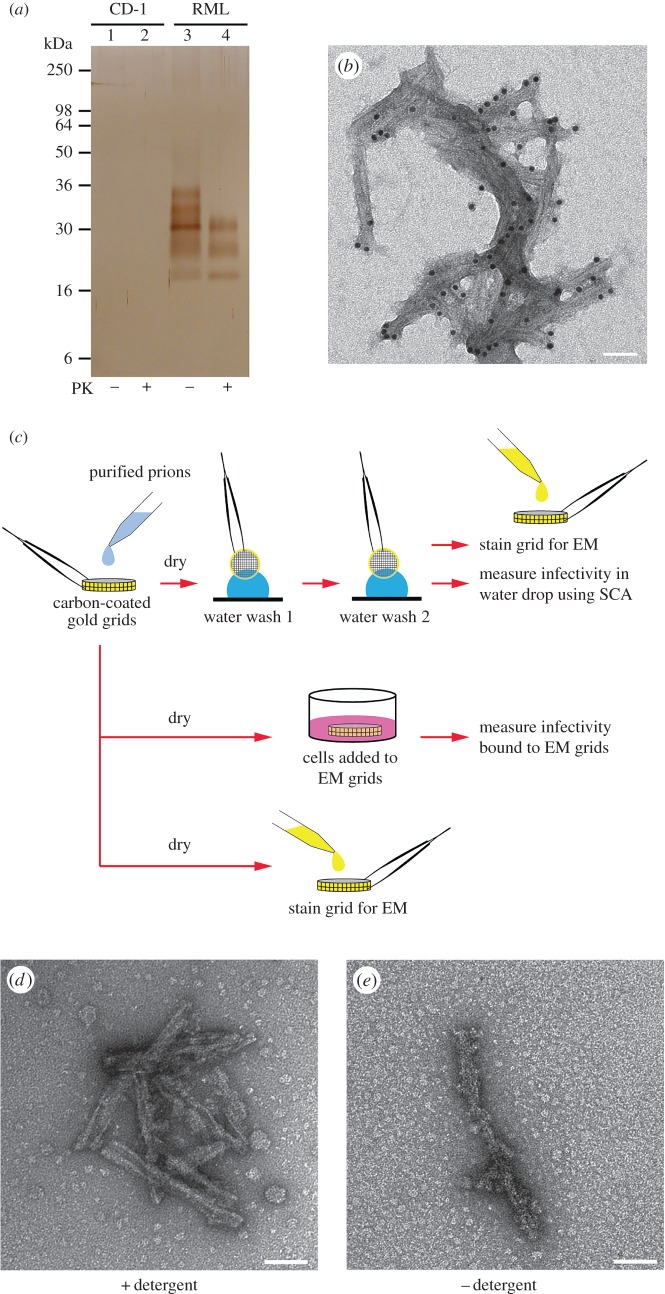
*Ex vivo* PrP rods are physically associated with prion infectivity. (*a*) Silver-stained 16% SDS-PAGE gel of RML prion preparations purified with (+) or without (–) proteinase K (PK) digestion compared to equivalent fractions from uninfected normal CD1 mouse brain. (*b*) EM image of an RML prion preparation purified without PK digestion. PrP rods (shown here immunogold-labelled with an anti-PrP monoclonal antibody) are the only visible protein structures. (*c*) Schematic of novel cell culture methods (Scrapie Cell Grid Assay, SCGA) used to measure prion infectivity bound to the surface of EM grids. (*d*) EM image of PrP rods (RML strain) observed when purified prion samples are dried onto the microscope grid without washing with water. Visible background objects are non-protein buffer artefacts (that include detergent micelles). Purified prions bind tightly to grids with less than 5% of the applied infectivity removed from dried grids by successive water washes; see the electronic supplementary material, table S1. The SCGA reported prion infectivity titre with an efficiency equivalent to, or better than, measuring prions in solution, see the electronic supplementary material, table S2. (*e*) EM image of PrP rods (RML prion strain) isolated from brain without the use of detergent. Scale bars, 50 nm.

Central to understanding prion infectivity is the ability to determine the structure of assemblies unequivocally known to represent the infectious state and to correlate morphologies with infectivity [1,2,18,19]. Although electron microscopy (EM) has been used for more than 30 years to investigate purified prion preparations, none of the observed EM structures to date have been definitively correlated with prion infectivity. Indeed, it is currently unknown whether prions adhere efficiently to the support film of EM grids. To establish that the PrP structures we observe by EM are directly associated with prion infectivity, we applied aliquots of highly purified mouse prions [10] (RML strain; figure 1*a*,*b*) to individual carbon-coated gold EM grids and measured surface bound infectivity in cell culture using a novel method that we term the Scrapie Cell Grid Assay (SCGA; figure 1*c*). We found that purified prions bind avidly to carbon-coated EM grids (less than 5% of the applied infectivity was removed from dried grids by successive water washes; electronic supplementary material, table S1), and remarkably the SCGA reported prion infectivity titre with an efficiency equivalent to, or better than, measuring prions in solution (electronic supplementary material, table S2). We conclude that prions bind to EM grids with near 100% efficiency and that their infectivity is not significantly altered once bound to the grid surface. These findings are reminiscent of the highly efficient binding of prions to stainless steel wires that has been documented previously [20–22]. Importantly, on EM grids on which the entire sample had been allowed to dry, PrP rods remain the only visible protein structures (figure 1*d*). To exclude the possibility that smaller PrP assemblies [6] (that are either too small or sparsely populated to be observed by EM) carried the infectivity, we fractionated the purified prion samples by filtration and similarly analysed fractions by EM and in cell culture. We found that less than 4% of the starting infectivity and PrP was recovered in filtrates from 0.1 µm pore-sized membranes. If smaller PrP assemblies of the size and specific infectivity reported by Caughey and colleagues [6] were present in our PrP rod preparations we would have expected these to have traversed the 0.1 µm-sized pores of the filter. However, we found no evidence for these in our preparations. When examined by EM the filtrates showed only occasional short PrP rods with morphology congruent with those retained by the filter. Collectively, these findings firmly establish a tight physical association between prion infectivity and the PrP rods observed by EM.

### 2.2. Prion protein rods are present in infected brain

**Figure 2.**
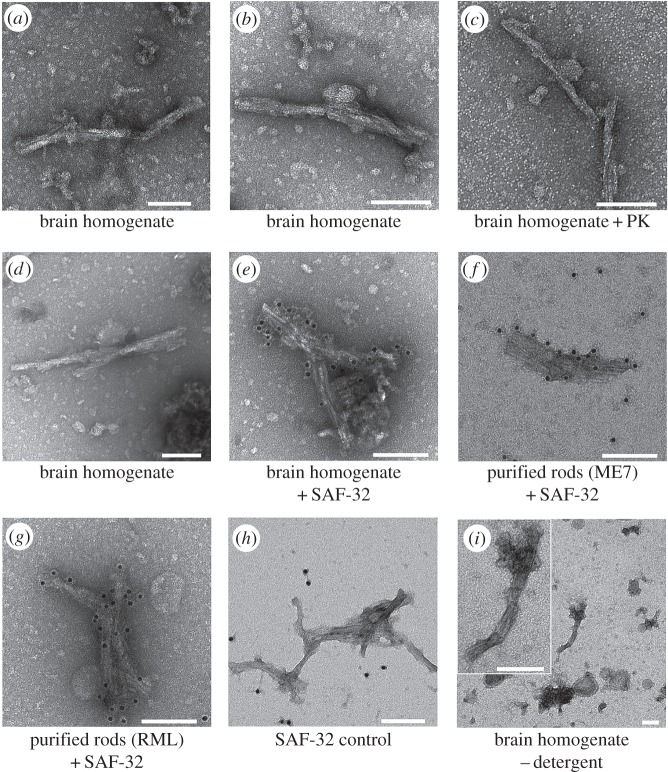
Visualization of PrP rods in crude RML brain homogenate. In total, 10% (w/v) RML brain homogenate was diluted twofold or 100-fold into 2% (w/v) sarkosyl (either lacking or containing 200 µg ml^−1^ PK) and incubated for various time periods (between 2.5 and 60 min). At the end of the incubation period, samples were centrifuged at 16 100*g* for 5 min. The supernatant was discarded and the pellets resuspended in TBS to one-third of the volume of the starting brain homogenate and immediately applied to EM grids. PrP rods were visualized by negative stain EM and their identity confirmed by immunogold labelling using anti-PrP monoclonal antibody SAF-32. The times reported include centrifugation time and the time taken to load EM grids. (*a*) RML brain homogenate diluted twofold in sarkosyl for 10 min. (*b*) RML brain homogenate diluted 100-fold in sarkosyl for 10 min. (*c*) RML brain homogenate diluted twofold in sarkosyl containing 200 µg ml^−1^ PK for 20 min. (*d*) RML brain homogenate diluted twofold in sarkosyl for 20 min (no PK). (*e*) Immunogold-labelled PrP rods in sarkosyl-treated RML brain homogenate. (*f*) Immunogold-labelled PrP rods in purified ME7 prion samples. (*g*) Immunogold-labelled PrP rods in purified RML prion samples. (*h*) A control experiment showing the specificity of the immunogold labelling method. Labelling of purified PrP rods (RML strain) is abolished when a 100-fold molar excess of recombinant mouse PrP is included with the SAF-32 primary antibody. (*i*) PrP rod isolated from RML brain homogenate without detergent. Scale bars, 100 nm.

The PrP rods in our purified mouse prion preparations have a gross morphology that closely resembles the prion rods seen in preparations of purified hamster prions isolated by Prusiner and colleagues [1,11]. These were considered artefactual and attributed to *in vitro* formation during purification of prions from brain by a process requiring both detergent and limited proteolysis of pathogenic PrP (PrP^Sc^) [17]. We sought evidence for such an *in vitro* assembly process by directly applying detergent solution (2% (w/v) sarkosyl in D-PBS) to crude 10% (w/v) RML brain homogenate and visualizing the samples by EM. In the absence of detergent, the heterogeneity of brain homogenate obscures visualization of PrP rods. However, exposure of brain homogenate to detergent for as little as 10 min readily facilitated visualization of PrP rods (figure 2*a*,*b*) with dimensions similar to those seen in highly purified samples. Importantly, the number and morphology of the PrP rods appeared to be the same regardless of the time of exposure of brain homogenate to detergent (from 10 to more than 60 min) or if brain homogenate was diluted 100-fold into detergent solutions in such reactions (figure 2*b*). Although EM is not a quantitative method (which precludes formal statistical analyses of PrP rod numbers), such apparent lack of time or protein concentration dependence does not support an *in vitro* assembly process as the source of the PrP rods unless this is extremely efficient and occurring on very short time scales (less than 10 min). To investigate this, we included a high concentration (200 µg ml^−1^) of proteinase K (PK) in the detergent solution and repeated the time course experiments. Despite the protease being present from the moment detergent and brain homogenate were mixed, we saw no detectable impact on our ability to observe the rods or any noticeable change in their morphology (compare figure 2*c* with figure 2*a*,*b*,*d*–*h*). Importantly, at this concentration, PK is sufficiently active to destroy PrP^C^ in normal mouse brain homogenate within 5 min [13]. Thus, if an assembly process were occurring on very short time scales, it would have to involve virtually no structural rearrangement of PrP conformation on addition of protein monomers to the rod, as any accessible scissile bonds should be targeted by the protease. Our findings also do not support detergent-facilitated assembly of the rods from smaller, protease-resistant, beta-sheet-rich PrP multimers. Such precursors were not seen by EM in our immunogold labelling experiments and again the kinetics of any assembly reaction would be critically dependent upon protein concentration. On the basis of these data, we considered that detergent serves to facilitate the observation of pre-existing PrP rods in brain homogenate rather than to catalyse their formation.

**Figure 3.**
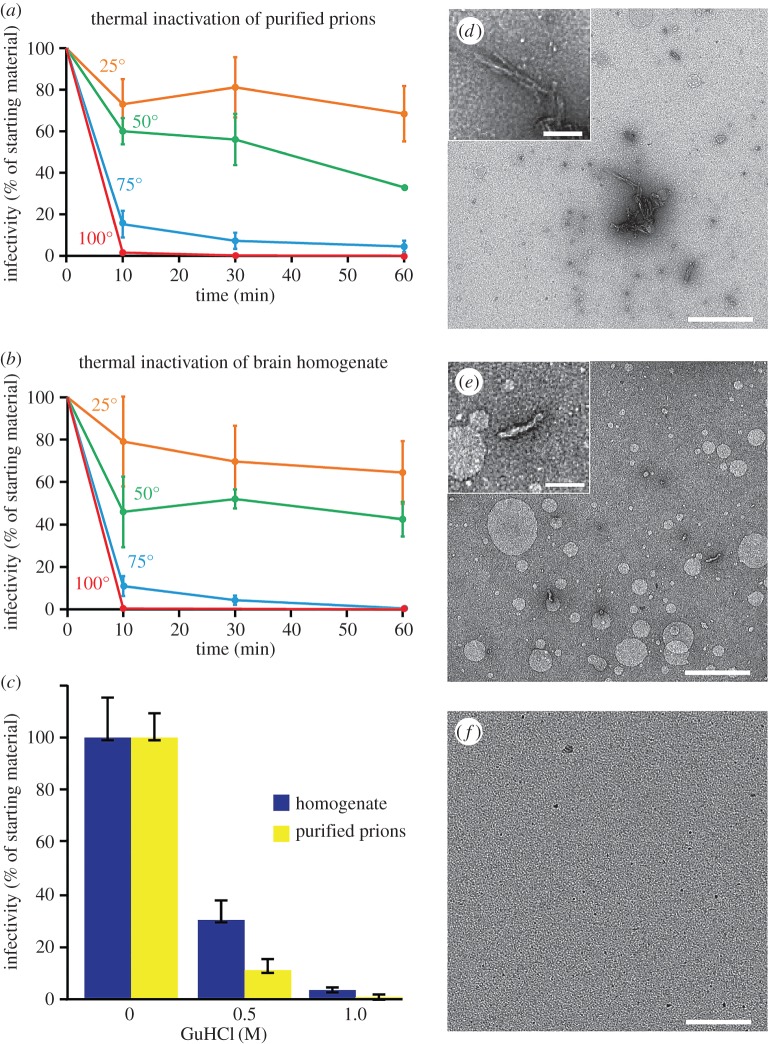
Heat and chemical inactivation of prions. Replicate aliquots of purified RML prions or RML brain homogenate were heated at different temperatures for defined times or incubated with different concentrations of guanidine hydrochloride (GuHCl) for 30 min and the infectivity in the samples measured using the Scrapie Cell Assay and expressed as a percentage (mean ± s.e.m., *n* = 3) of that present in the starting preparation. Samples were also analysed by immunoblotting (see the electronic supplementary material, figure S1). (*a*,*b*) Thermal inactivation profiles, purified prions (*a*), brain homogenate (*b*). (*c*) GuHCl inactivation. (*d*–*f*) EM images of purified prions incubated for 30 min at 25°C (*d*), 50°C (*e*) or 100°C (*f*), showing thermal denaturation of PrP rods. PrP rods were more sensitive to digestion with PK after heat denaturation of prion infectivity at 100°C compared to 25°C (see the electronic supplementary material, figure S1). Scale bars, main panels 500 nm, magnified insets 100 nm.

Further experiments strongly supported this idea. We were able to isolate PrP rods from brain without using detergent (figures 1*e* and 2*i*) and found that they have a similar morphology to those present in highly purified samples. Consistent with this finding, we also found that the thermal and chemical inactivation profiles of prion infectivity in brain homogenate and purified prion preparations were closely similar (figure 3*a*–*c*). PrP rods (figure 3*d*,*e*) were no longer detectable by EM in samples in which infectivity had been denatured, either in purified prion samples (figure 3*f*) or in brain homogenate. Heat denaturation of prion infectivity at 100°C (which typically reduces prion titre by more than 100-fold when measured by rodent bioassay [23,24]) could be correlated with a conformational change of PrP within the rods as evidenced by increased sensitivity of the protein to digestion with PK (electronic supplementary material, figure S1). The similarity of the thermal and chemical inactivation profiles of prion infectivity in brain homogenate and purified samples clearly suggests the destruction of the same infectious structures in both preparations. Based upon our collective data, we conclude that the PrP rods observed in our purified prion samples originate from the brain and are not assembled *in vitro*. Demonstration that PrP rods are intrinsically infectious and originate *in vivo* firmly establishes the importance of understanding their structure.

### 2.3. Aggregates of prion protein rods behave as discrete infectious particles

**Figure 4.**
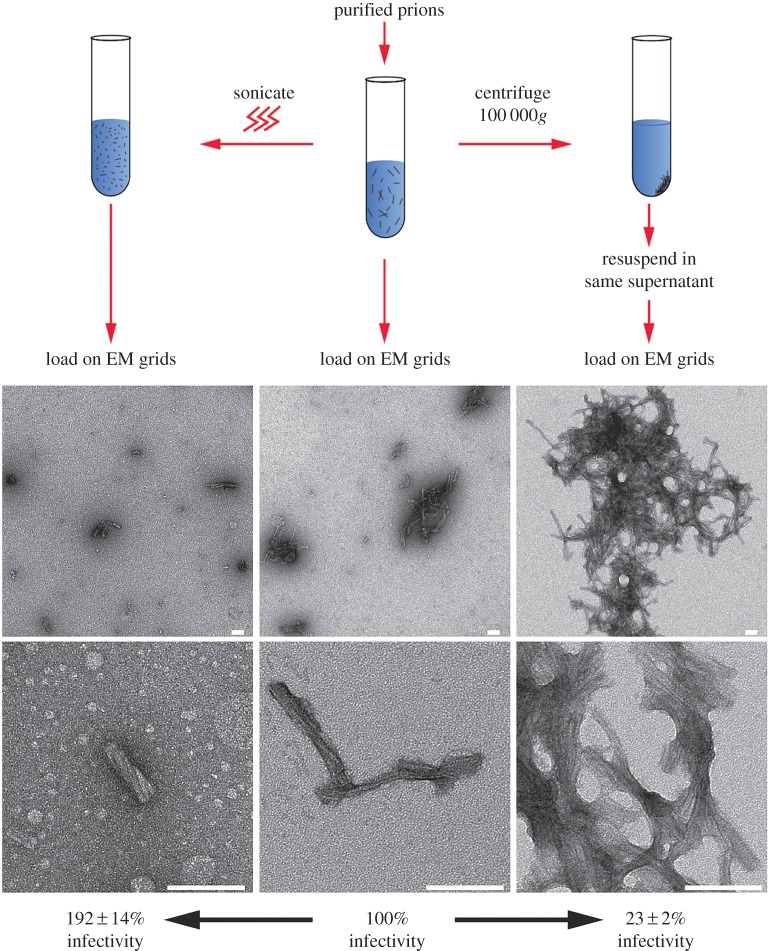
Aggregates of PrP rods act as discrete infectious particles in cell culture. Replicate aliquots of purified RML prions were either sonicated or centrifuged to alter the aggregation state of PrP rods and compared to untreated samples by negative stain EM. Infectivity titre of the treated samples was determined using the Scrapie Cell Assay and expressed as a percentage of that present in the untreated sample (mean ± s.d., *n* = 3). Sonication generates a greater number of small dispersed aggregates of PrP rods and higher prion titre, whereas centrifugation produces a reduced number of larger sized aggregates and lower prion titre. Scale bars, 100 nm.

All preparations of purified prions have a large ratio of PrP molecules per infectious unit in rodent bioassay [6,10,11,25] and in cell culture [10]. Differences in the length of infectious PrP rods (in which the propagating ends of the rod can be considered the infectious entity [2,26,27]) can readily accommodate variance in specific prion infectivity with respect to PrP monomers. Since the rods aggregate with non-uniform distributions on the grid, their concentration could not be estimated from the images. Nevertheless, the dimensions of the PrP rods could be readily estimated, as could their aggregation state. The rods appeared relatively consistent in width (approx. 21 nm) and although their length varied (50–350 nm), most were between 100 and 200 nm long (electronic supplementary material, table S3). The greatest variation that we observed was the number of PrP rods per aggregate. Individual aggregates could contain from five to several hundred individual PrP rods and we rarely observed PrP rods in isolation. Therefore, we considered that PrP rod aggregates might act as discrete infectious particles in cell culture. To test this, identical aliquots of purified prions were treated to re-distribute the PrP rods in the sample into a greater or smaller number of aggregates, after which infectivity was measured in the Scrapie Cell Assay and the samples visualized by EM (figure 4). Findings from this experiment were unequivocal. Sonicated samples had a greater number of small dispersed aggregates of PrP rods and had a higher specific infectivity than untreated samples, whereas centrifuged samples had a lower number of much larger PrP rod aggregates and lower specific infectivity (figure 4). While sonication caused fragmentation of some of the PrP rods to produce shorter lengths (thus increasing the number of ends) as well as altering aggregate size and number (figure 4), centrifugation only caused alterations to aggregate size and number. Thus, without adding to, or removing, anything from the samples we established that changing the number of aggregates into which the PrP rods are distributed changes the number of infectious units available to cells at inoculation. These findings demonstrate that PrP rods are the constituents of discrete infectious particles whose size can be altered. The ability to alter specific prion infectivity simply by centrifuging the sample to produce larger aggregates of the rods indicates that such effects on bioavailability must be considered when characterizing purified prion preparations. These data also highlight the power of cell culture infectivity assays to gain new insight into the physical basis of prion infectivity as the variance in the specific infectivity that we observed in our samples (approx. eightfold) lies within the typical error range associated with measuring prion infectivity titre in rodent bioassays (typically mean ± 0.5 log) [28].

### 2.4. Three-dimensional structure of prions

**Figure 5.**
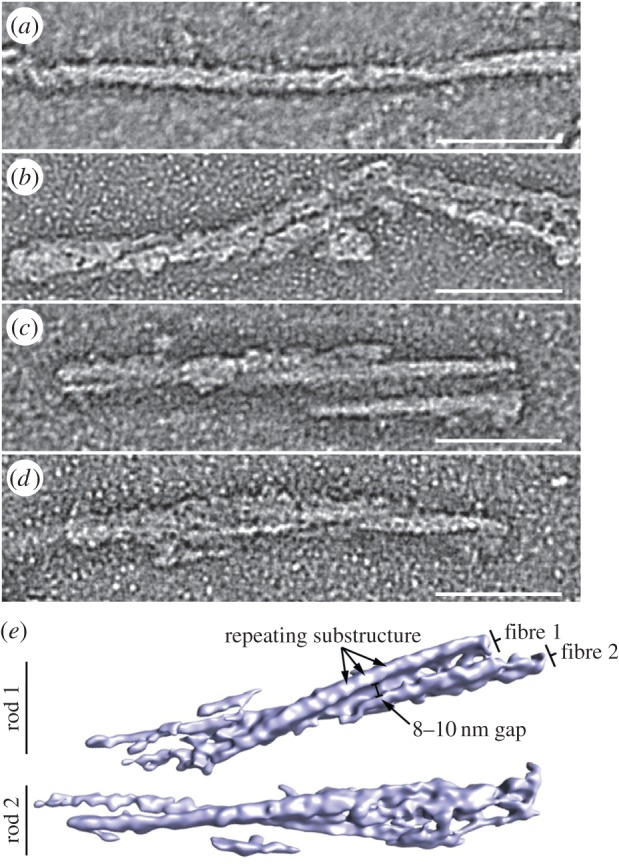
Three-dimensional reconstruction by electron tomography of prions reveals structures distinct from non-infectious recombinant PrP fibrils. (*a*–*d*) Sections through negative stain electron tomography reconstructions. (*a*) Non-infectious recombinant PrP fibril compared with infectious *ex vivo* PrP rods from (*b*) RML-infected CD1 mouse brain, (*c*) ME7-infected C57Bl/6 mouse brain and (*d*) RML-infected C57Bl/6 mouse brain. Dimensions of the PrP rods are provided in the electronic supplementary material, table S3. (*e*) An isosurface view of a tomographic reconstruction (see the electronic supplementary material, movie S1) of two individual ME7 prion rods purified from C57BL/6 mouse brain showing each rod to be composed of two fibres twisted round one another separated by a distinct gap 8–10 nm in width. The repeating substructure is less evident than in the density sections, after the filtering needed for isosurface representation. Scale bars, 50 nm.

**Figure 6.**
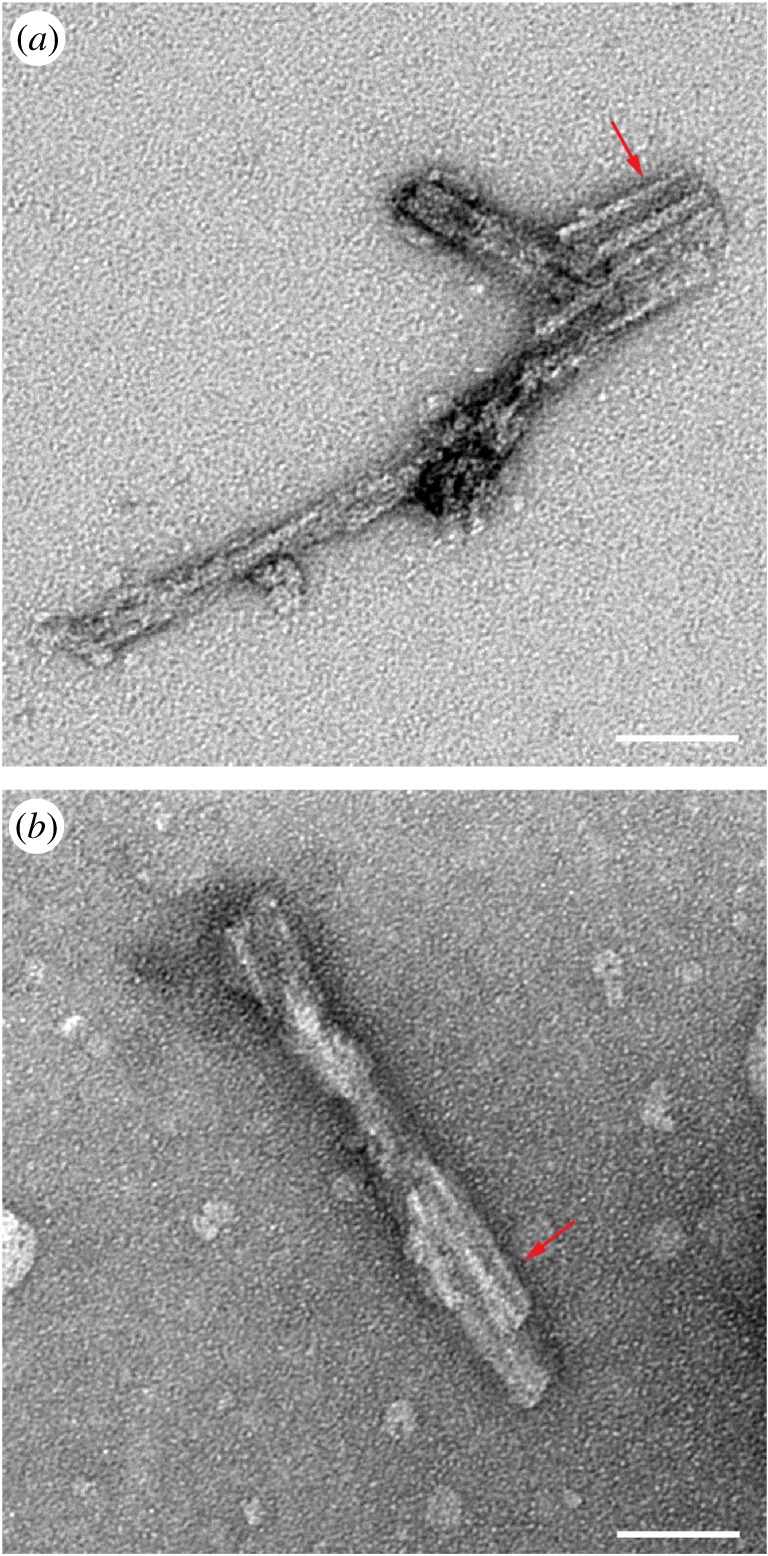
*Ex vivo* infectious PrP rods have a common building block regardless of prion strain type. Common structural features are recognizable in two-dimensional negative stain images of PrP rods isolated from (*a*) RML prion-infected CD1 mouse brain and (*b*) Sc237 prion-infected hamster brain (red arrows) showing paired PrP fibres with a double helical substructure, separated by an 8–10 nm gap. Both images show a typical view of multiple PrP rods in an aggregate (detergent micelles can be observed in the background). Scale bar, 50 nm.

Having provided compelling physical evidence linking *ex vivo* PrP rods with prion infectivity and showing that discrete aggregates of PrP rods appear to behave as infectious particles in cell culture, we analysed their three-dimensional structure by electron tomography of negatively stained samples. All the prions examined showed a common structure. The rods contained a pair of short, intertwined fibres, each with a double helical repeating substructure, separated by a distinct gap 8–10 nm in width (figure 5; electronic supplementary material, table S3). The structure of this twisted assembly is difficult to discern in EM images, which are two-dimensional density projections. However, the twist of the paired fibrils as well as a twisted two stranded structure within each fibril become apparent when the three-dimensional structure of the assembly is resolved by tomography (electronic supplementary material, movie S1). The hierarchical assembly of paired fibrils into PrP rods has not previously been described in three dimensions [6,11,17,29,30], nor has there been a three-dimensional structural comparison of infectious and non-infectious PrP assemblies. Our three-dimensional analysis strongly suggests that all *ex vivo* infectious PrP rods have a common building block comprising paired fibres with a double helical substructure regardless of prion strain type (figure 5; electronic supplementary material, movie S1). On the basis of our tomograms, these structural features can now be readily recognized in our two-dimensional images of PrP rods (consistently in multiple preparations and thousands of rods visualized) [10] (figure 6) and in earlier images of PrP rods isolated from hamster [17] or mouse brain [29] by others. While structural differences that distinguish prion strains may become apparent with higher resolution methods (such as cryo-tomography and subtomogram averaging), the architecture of the PrP rods now provides a basis for explaining the distinctive physico-chemical properties of prions in comparison to non-infectious PrP fibrils.

PrP fibrils generated by us from recombinant PrP show no infectivity in either cell culture or rodent bioassay, even when assayed at high concentrations or after sonication to produce more fibril ends (electronic supplementary material, figure S2 and table S4) [31]. In contrast to the structural complexity of PrP rods, they have a simpler construction [32] (figure 5*a*). These recombinant PrP fibrils resemble the individual fibres present in *ex vivo* PrP rods, but they do not assemble to produce intertwined paired fibres (figure 5; electronic supplementary material, table S3). The recombinant fibrils have a double helical arrangement of two protofilaments with a 60 Å globular repeat [32], and are very long and somewhat wider than the individual fibres in the *ex vivo* PrP rods. Recent structural modelling of non-infectious recombinant PrP fibrils generated through prion-seeded real-time quaking-induced conversion (RT-QuIC) reactions suggested that PrP rods are single protofilaments [30], but this proposal is incompatible with the three-dimensional architecture of the PrP rods that we describe here. Hierarchical assembly of PrP into a paired fibre structure (figure 5*b*–*e*) appears to be a key feature of infectious prions. This arrangement may contribute significantly to the remarkable resistance of prions to degradation, which is likely to be crucial to their natural transmissibility, for example by dietary exposure. Whereas non-infectious recombinant PrP fibrils have a highly exposed surface, the paired fibres within the PrP rod may be less accessible to proteases. Moreover, while recombinant PrP fibrils are composed solely of non-glycosylated PrP, *ex vivo* PrP rods are composed of defined ratios of di-, mono- and non-glycosylated PrP [10]. The characteristic 8–10 nm gap between the two fibres of repeating structure in the rods (figure 5*b*–*e*; electronic supplementary material, movie S1) suggests a possible location for some of the N-linked glycans that may contribute to the stability of the assembly, and as a consequence its infectivity. Such an arrangement of the glycans may protect a significant fraction of the protein surface and suggests a plausible steric basis for generating the highly consistent PrP glycoform ratios that characterize different prion strains [10,33–36]. Regarding glycan accessibility, prolonged incubation of RML PrP rods with neuraminidase, a range of endoglycosidases or PNGase F had no discernible effect on the morphology of the rods when viewed by EM or on the mobility of PrP glycoforms when viewed on immunoblots (2015, our unpublished observations). These findings suggest that the N-linked glycans are buried within the structure of the rod, but further experiments are required to confirm this.

## 3. Discussion

Progress in understanding prion structure has been severely hindered by the difficulty of isolating relatively homogeneous prion particles from affected tissue and unequivocally correlating infectivity with composition and structure. Here, using new and highly effective methods for prion isolation [10] together with sensitive cell culture infectivity assays we show that PrP rods in our preparations originate *in vivo* and are intrinsically infectious. Once isolated, aggregates of the rods behave as discrete infectious particles in cell culture. Electron tomography shows that PrP rods have a common hierarchical structure which is very different to the individual, long, non-infectious PrP fibres generated *in vitro* from recombinant PrP. This distinctive three-dimensional structure now provides a new basis for understanding prion infectivity and the features that differentiate prions from non-infectious protein assemblies. Determining the three-dimensional structure of the rods at a higher resolution is now clearly required to establish the location of the N-linked glycans which may be involved in linking the fibres. In this regard, biologically relevant, high-affinity glycan : glycan interactions have recently been reported [37]. Systematic generation of prions from recombinant PrP remains an extremely challenging research goal [31]. However with more detailed knowledge of the rod structure, it may become possible to engineer single recombinant PrP fibres into a paired assembly to generate high-titre synthetic prions.

Presently, the mechanism of assembly of prions *in vivo* remains to be determined. To date, we have been unable to separate the individual fibres in the PrP rods by chemical or physical means while still retaining infectivity. We have also not observed single fibres in our purified preparations, strongly suggesting that the two fibres co-assemble during prion formation. Clearly, if this is the case, the rods must elongate *in vivo* and the presence of PrP rods of different lengths in brain homogenate (together with variable association with lipids) may underlie the wide distribution of prion infectivity that is seen when brain homogenate is fractionated in density gradients [5,7–9]. Resolving the structural relationship between small-sized infectious particles in brain homogenate with that of the larger infectious PrP rods that we describe here remains an important goal, as the smaller particles appear to account for a substantial proportion of the total infectivity in brain homogenate [5–9]. On the basis of this work, it seems highly likely that the small-sized infectious particles in brain homogenate are simply truncated PrP rods. Testing this hypothesis however remains highly challenging due to the difficulty of isolating homogeneous preparations of the small-sized particles from brain.

## 4. Material and methods

### 4.1. Research governance

Work with prion-infected samples was conducted in microbiological containment level 3 or level 2 facilities with strict adherence to safety protocols.

### 4.2. Prion sources

In total, 10% (w/v) brain homogenates from terminally affected CD1 mice propagating the RML prion strain (I6200), from terminally affected C57Bl/6 mice propagating either the RML (I14051) or ME7 (I14050) prion strain, or from terminally affected Syrian hamsters propagating Sc237 prions (I9200) were prepared as described previously [10]. Normal 10% (w/v) brain homogenates from uninfected CD1 mice (I10340 or I14040) were used as control samples. Homogenates were stored in aliquots at −70°C.

### 4.3. Prion purification

Comprehensive details of the purification method have been described previously [10]. We used purified P4 fractions prepared without PK digestion. The method produces a recovery of approximately 10% of the prions present in the starting 10% (w/v) brain homogenate so that resuspension of the purified P4 pellet fraction in buffer at one-tenth of the volume of the 10% (w/v) brain homogenate from which it was derived produces prion preparations whose infectivity titre matches that of the starting 10% (w/v) brain homogenate [10]. Subsequent concentration of the purified prions or buffer exchange was achieved by centrifugation at 16 100*g* for 30 min and resuspension of the pellet fraction into the desired volume and buffer of choice. SDS-PAGE, silver staining and PrP immunoblotting (using anti-PrP monoclonal antibody ICSM 35; D-Gen Ltd, London, UK) was performed using established procedures [10,38].

### 4.4. Filtration experiments

Twenty-five microlitre aliquots of purified RML prions (prion titre approx. 4× that of 10% (w/v) RML brain homogenate) in 20 mM Tris, 150 mM NaCl pH 7.4 (TBS) containing 0.1% (w/v) sarkosyl were thoroughly mixed with a pipette tip and then sonicated briefly in a Sonicator 3000 (Misonix) at 40 W at 4°C for a total of 60 s (2× 30 s intervals) to disrupt large PrP aggregates. Twenty microlitre aliquots were added to pre-wetted 0.1 µm Ultrafree-MC centrifugal PVDF filter units (Millipore, Cat # UFC30VV25) and centrifuged at 12 000*g* for 30 s. The filtrate was isolated and the retentate resuspended in 20 µl of TBS buffer containing 0.1% (w/v) sarkosyl. Samples (including the sonicated starting material) were analysed in the Scrapie Cell Assay (SCA) and by EM.

### 4.5. Standard Scrapie Cell Assay

Prion samples were diluted appropriately (typically either 1 : 1000 or 1 : 10 000) in OptiMEM or MEM (Invitrogen) and infectivity quantified in the SCA using PK1/11 cells for RML prions or LD9 cells for ME7 prions as previously described [10,12]. After exposing the cells to prion samples for 3 days, cells were split 1 : 8 into fresh cell culture media and grown back to confluence. Two additional 3 day splits were performed before transferring cells to ELISPOT (Multi Screen Immobilon-P, Millipore, UK) plates to quantify the number of cells containing PK-resistant PrP (spot numbers). The SCA was calibrated by concomitant analyses of a reference 10% (w/v) RML brain homogenate of known intracerebral mouse LD50 prion titre (I6200; prion titre of 10^7.3±0.5^ (mean ± s.d.) intracerebral LD50 units ml^−1^) determined from six endpoint titrations in Tg20 mice [10]. All infectivity data (spot numbers) were correlated with the reference RML brain homogenate and infectivity expressed as the equivalent number of intracerebral mouse LD50 units present in the purified prion samples.

### 4.6. Measuring prion infectivity in successive water washes of electron microscopy grids

Two microlitres of purified RML prions (prion titre approx. 1× that of 10% (w/v) RML brain homogenate) were loaded onto three separate glow-discharged 300 mesh carbon-coated gold grids (Agar Scientific Ltd, Cat # S160A3) and left to dry completely. The grids were then drawn successively through two 20 µl drops of water after which any excess water on the grid was removed with a pipette tip and pooled with the second water drop. In total, 2.5 µl aliquots were withdrawn from each of the water drops and diluted into OptiMEM tissue culture media and tested for their infectivity using the Scrapie Cell Assay. The infectivity titre of 2 µl aliquots of the purified RML prion sample used to load the grids was concomitantly determined, enabling the infectivity in the water washes to be expressed as a percentage of that applied to the grids. Three grids loaded with uninfected 10% (w/v) CD1 brain homogenate were performed in parallel as a negative control. All grids from these experiments were also visualized by negative stain EM to confirm that PrP rods were bound to carbon and that the carbon remained intact and was still attached to the grids. Any grids from which carbon had detached were not included in infectivity analyses.

### 4.7. Scrapie Cell Grid Assay

To measure the infectivity of samples bound to EM grids, 3 µl of purified prions (prion titres ranging from 0.01× to 1× that of 10% (w/v) RML brain homogenate) were loaded onto three separate glow-discharged 300 mesh carbon coated gold grids (Agar Scientific Ltd, Cat # S160A3) and left to dry completely. Grids were transferred into a 20 µl drop of cell culture medium inside a 24-well tissue culture plate and 2 ml of PK1/11 cell suspension at 50 cells µl^−1^ in OFCS (OptiMEM, 10% FCS, penicillin and streptomycin) added to the grids so that a total of 100 000 cells were added per well. Cells were incubated for 3 days then harvested by washing the grid surface twice with 2 × 100 µl of OFCS medium and transferred to a clean 24-well plate. All 200 µl washes from three identical replicate grids were pooled (600 µl total volume), cells adjusted to a density of 16 cells µl^−1^ with OFCS media, then 4000 cells per well (250 µl) transferred to a new 96-well plate. After 4 days incubation, cells were resuspended then split at a 1 : 8 dilution into fresh OFCS media and passaged in a standard Scrapie Cell Assay format, in which the cells were grown to confluence and split 1 : 8 three times before transferring samples of the cells to ELISPOT plates for analysis of the number of cells containing PK-resistant PrP. Infectivity of prion-containing samples was calculated by correlation with a serial dilution of 10% (w/v) brain homogenate of known infectious titre and expressed in LD50 ml^−1^. To determine the efficiency with which the SCGA reported prion titre, replicate aliquots of the purified prion samples were also concomitantly measured using the standard Scrapie Cell Assay format (with samples diluted 1 : 10 000). In addition, 3 µl replicate aliquots of the purified prion samples were added to a 20 µl drop of media in a 24-well tissue culture plate then 2 ml of PK1 cell suspension at 50 cells µl^−1^ in OFCS added. Cells were incubated for 3 days, after which each confluent well was resuspended, the cells adjusted to a density of 16 cells µl^−1^ with OFCS media and 4000 cells per well (250 µl) transferred to a new 96-well plate. Thereafter, cells were processed identically to those harvested from EM grids. Uninfected CD1 brain homogenate was examined in parallel in all the infectivity assay formats to act as a negative control.

### 4.8. Visualizing prion protein rods in crude brain homogenate using detergent

Our experience with purified RML prion samples showed that application of greater than or equal to 10^5^ intracerebral LD50 units to EM grids is required in order to readily visualize PrP rods. Experiments with brain homogenate were therefore designed with this knowledge. Fifteen microlitres of 10% (w/v) RML brain homogenate was diluted twofold or 100-fold with 2% (w/v) sarkosyl (prepared in D-PBS) and incubated at room temperature for various time periods between 2.5 and 60 min, after which the samples were centrifuged at 16 100*g* for 5 min. Supernatants were discarded and the pellets resuspended in 4 µl of TBS buffer then immediately loaded onto EM grids and subsequently visualized by negative stain EM. Further time course experiments were performed in the presence of PK (Merck, Cat # 70663) at 200 µg ml^−1^ from the time of addition of detergent to the brain homogenate. PK activity was inhibited prior to centrifugation of the sample by the addition of 4-(2-aminoethyl)-benzene sulfonyl fluoride (AEBSF; Melford, Cat # T2147) to 1 mM final concentration.

### 4.9. Isolation of prion protein rods from brain homogenate in the absence of detergent

Two hundred microlitres of D-PBS plus 0.8 µl of Benzonase (25 U µl^−1^) were added to 200 µl of 10% (w/v) RML brain homogenate, mixed thoroughly and then incubated at 37°C for 30 min with gentle agitation. Optiprep was added to a final concentration of 35% (v/v) and mixed thoroughly, after which the sample was centrifuged at 16 100*g* for 90 min at 37°C. The lipid surface layer was removed and retained. Forty microlitre aliquots of this fraction were mixed with 160 µl of methanol and incubated for 1 h at 25°C. Samples were then centrifuged at 16 100*g* for 30 min and the pellets resuspended in 5 µl of water, after which 3 µl was loaded onto EM grids. Samples produced by this method have a heterogeneous background when visualized by EM. Much of the imaged surface is obscured by micellar structures and extensive examination of the grids is required to find PrP rods.

### 4.10. Heat denaturation of prions in brain homogenate and purified samples

Ten microlitres of purified RML prions (prion titre approx. 1× that of 10% RML brain homogenate) in TBS buffer containing 0.1% (w/v) sarkosyl were transferred to Protein LoBind tubes and incubated at 25, 50, 75 and 100°C for 10, 30 or 60 min with gentle agitation. In parallel, 10 µl aliquots of 10% (w/v) prion-infected brain homogenates (in D-PBS) were incubated under the same conditions. At the defined time points, samples were pulse centrifuged to recover the sample to the bottom of the tube and immediately frozen on dry ice. Aliquots of each sample were analysed for infectivity in the Scrapie Cell Assay and examined by EM. For analysis by EM, 2.5 µl of the samples were loaded onto EM grids. To assess the relative PK sensitivity of the material incubated at different temperatures, aliquots of the brain homogenate or purified prion samples were digested with final concentrations of 50 or 10 µg ml^−1^ PK, respectively, for 1 h at 37°C. Digestion was terminated by the addition of AEBSF to a final concentration of 1 mM after which samples were immediately processed for PrP immunoblotting.

### 4.11. Guanidine hydrochloride denaturation of prions in brain homogenate and purified samples

Ten microlitre aliquots of purified RML prions (prion titre approx. 2.5× that of 10% RML brain homogenate) or aliquots of 10% (w/v) RML brain homogenate (in D-PBS) were dispensed into Protein LoBind tubes (Eppendorf) and centrifuged at 16 100*g* for 30 min (or 10 min for homogenate). The supernatants were removed and the pellets resuspended in 20 mM Tris buffer pH 7.4 containing 0.1% (w/v) sarkosyl and either 0, 0.5 or 1.0 M GuHCl (Sigma-Aldrich, Cat # G3272) in a final volume of 10 µl. In addition, replicate controls were set up in parallel using 20 mM Tris buffer pH 7.4 containing 0, 0.5 and 1 M NaCl. Following gentle agitation at 25°C for 30 min, reactions were stopped by freezing the samples on dry ice. Aliquots of each sample were analysed for infectivity in the Scrapie Cell Assay, examined by EM or processed for PrP immunoblotting before or after digestion with PK as described above for heat treated samples. For infectivity measurement in the Scrapie Cell Assay, samples were diluted either 1 : 1000 or 1 : 10 000 in tissue culture medium so that the final concentration of GuHCl was below 1 mM to prevent toxicity to the cells.

### 4.12. Sonication and ultracentrifugation of purified prions

One hundred microlitres of purified RML prions (prion titre approx. 1× that of 10% RML brain homogenate) were transferred to a Sonicator 3000 (Misonix) at 4°C. Samples were sonicated at 40 W for a total of 10 min using 30 s bursts with 60 s intervals between bursts then pulse centrifuged to recover the sample to the bottom of the tube. Concomitantly, identical 100 µl aliquots of the purified RML prions were centrifuged at 100 000*g* maximum in a TLA-110 rotor for 60 min using an Optima MAX-XP ultracentrifuge (Beckman Coulter) at 25°C. Resulting pellet fractions were gently resuspended back into the supernatant (within the same tube) using a pipette tip. Sonicated or centrifuged samples together with replicate aliquots of the untreated purified RML prions were analysed by EM, in the Scrapie Cell Assay and by PrP immunoblotting or ELISA [10] to determine the PrP content of the samples.

### 4.13. Growth of recombinant prion protein fibrils

Recombinant mouse PrP (*Prnp* allele a; amino acid residues 91–231) was converted into a β-sheet conformation [32,39] by incubation in 10 mM sodium acetate/10 mM tris-acetate buffer pH 8.0 containing 100 mM dithiothreitol (DTT) and 6 M GuHCl for 16 h, followed by dialysis in 10 mM sodium acetate/10 mM tris-acetate buffer pH 4 containing 1 mM DTT. Insoluble material was removed by centrifugation at 150 000*g* for 4 h. The supernatant was concentrated using Vivaspin 20 centrifugal concentrators. Subsequently, samples were adjusted with 10 mM sodium acetate/10 mM tris-acetate buffer pH 3.0 to give a final protein concentration of 1.2 mg ml^−1^, after which 100 µl aliquots were incubated for three to five months without agitation at 25°C in sealed 1.5 ml tubes as described previously [32]. Fibrils were harvested by centrifugation of the solutions at 13 000*g* for 45 min then pellets resuspended in 10 mM sodium acetate/10 mM tris-acetate buffer pH 3.0–50% of the original volume. To disrupt recombinant PrP fibrils (when required), samples were sonicated at 40 W in a Sonicator 3000 (Misonix) at 4°C for a total of 10 min using 30 s bursts with 60 s intervals between bursts. Samples were then either diluted 1 in 5 in 10 mM sodium acetate/10 mM tris-acetate buffer pH 3.0 and loaded onto EM grids or analysed for infectivity using the Scrapie Cell Assay.

### 4.14. Negative stain electron microscopy

Samples were loaded on EM grids within a class 1 microbiological safety cabinet in a microbiological containment level 3 laboratory. Three microlitres of purified prions (prion titre typically approx. 2.5× that of 10% RML brain homogenate) or recombinant PrP fibrils (diluted 1/5, approx. 0.5 mg ml^−1^) were loaded onto 300 mesh 3.05 mm carbon-coated copper grids (Electron Microscopy Sciences, Cat # CF300-Cu) that had been glow discharged for 40 s using an EMS 100× glow discharge unit (Electron Microscopy Sciences, USA). Typically, samples were adsorbed to the grid surface for 2 min after which excess solution was removed by blotting with grade 4 Whatman paper. Grids were then washed briefly by passing through two water drops then stained with 2% (w/v) uranyl acetate for 45 s, blotted and air dried. In some cases samples were completely dried on to the grids, in which case water washes were omitted and the grids then stained with uranyl acetate, blotted and air dried. Grids loaded with infectious samples were inserted into the microscope using a dedicated sample holder for mouse prions with strict adherence to risk assessment and microbiological containment level 2 safe working practice. Images were acquired on an FEI Tecnai T10 electron microscope (FEI, Eindhoven, NL) operating at 100 kV and recorded on a 1 k × 1 k charged couple device (CCD) camera (Gatan) at a nominal magnification of 44 000 with a pixel size of 3.96 Å.

### 4.15. Immunogold labelling of purified prions or prion protein rods in crude brain homogenate

Purified prion rods isolated from the equivalent of 0.5 ml of 10% (w/v) RML brain homogenate were labelled with anti-PrP monoclonal antibody SAF-32 (Bioquote Ltd, Cat # 189720) and goat anti-mouse IgG conjugated to 10 nm gold particles (Sigma-Aldrich, Cat # G7652) as described previously [10]. To label PrP rods in crude brain homogenate, an equal volume of 4% (w/v) sarkosyl was added to 100 µl 10% (w/v) RML brain homogenate followed by incubation at 37°C for 30 min. The sample was then centrifuged at 16 100*g* for 30 min. Pellets were resuspended in 100 µl of TBS containing 5% (v/v) glycerol and 0.1% (w/v) sarkosyl and passed through a 0.65 µm Ultrafree-MC Centrifugal PVDF filter unit (Millipore, Cat # UFC40DV25) to remove collagen fibres and intermediate filaments. Labelling of the filtrate fraction was then performed as described for purified prion samples [10].

### 4.16. Electron tomography

Samples were loaded onto glow-discharged 200 mesh carbon-coated copper finder grids (Electron Microscopy Sciences, Cat # CFLF200-Cu) and stained as described for negative stain EM. Single- and dual-axis tilt series were acquired using a dual-axis tomography holder (Fischione Instruments) on a Tecnai 12 electron microscope (FEI, Eindhoven, NL) operating at 120 kV. SerialEM [40] was used to collect the tilt series using a tilt range of –70° to +70° with 2° increments. Digital images were recorded on a 1 K Gatan Multiscan 794 CCD camera at a nominal magnification of 42 000× with a pixel size of 4.1 Å and a typical defocus of 0.7 µm. Volumes for 17 recombinant PrP fibrils were analysed from four tomograms (reconstructed from 2 single- and 2 dual-axis tilt series), volumes for 28 *ex vivo* PrP rods purified from RML-infected CD1 brain were analysed from eight tomograms (reconstructed from 3 single- and 5 dual-axis tilt series), volumes for 21 *ex vivo* PrP rods from ME7-infected C57Bl/6 brain were analysed from seven tomograms (reconstructed from 2 single- and 5 dual-axis tilt series), and volumes for 24 *ex vivo* PrP rods from RML-infected C57Bl/6 brain were analysed from four tomograms (reconstructed from 3 single- and 1 dual-axis tilt series). After use, the holder was decontaminated by plasma cleaning for 15 min (Fischione Instruments). Tomograms were reconstructed from the tilt series with IMOD v. 4.3 [41], using local patch tracking for alignment. The isosurface view (figure 5*e*) was rendered after Gaussian low-pass filtering and removal of isolated noise densities in CHIMERA (http://www.cgl.ucsf.edu/chimera/).

## Supplementary Material

Figures S1 and S2 and Tables S1-S4

## Data accessibility

Extended data are provided in the electronic supplementary material (tables S1–S4, figures S1 and S2 and movie S1).
