# Supplementary material for: Ex vivo mammalian prions are formed of paired double helical prion protein fibrils
Source: Open Biol. 2016 May 4;6(5):160035. doi: 10.1098/rsob.160035 (PMC4892434; doi:10.1098/rsob.160035)
Supplement: Figures S1 and S2 and Tables S1-S4 [file rsob160035supp1.pdf]

Supplementary Material for:

Ex vivo mammalian prions are formed of paired double helical PrP fibrils

Cassandra Terry, Adam Wenborn, Nathalie Gros, Jessica Sells, Susan Joiner, Laszlo L.P. Hosszu, M. Howard Tattum, Silvia Panico, Daniel K. Clare, John Collinge, Helen R. Saibil, Jonathan D.F. Wadsworth

Table of Contents

Supplementary Figure S1

Supplementary Figure S2

Supplementary Table S1

Supplementary Table S2

Supplementary Table S3

Supplementary Table S4

Caption for Supplementary Movie S1

Supplementary Figure S1

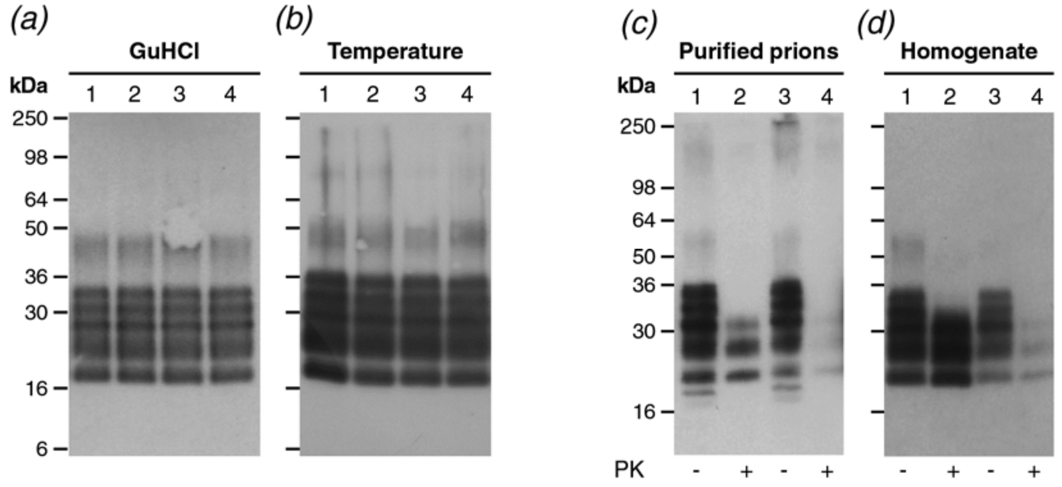

Supplementary Figure S1. PrP immunoblots of GuHCl and heat treated RML prions. (*a*) Lanes 1-4, purified RML prions treated with 0, 0.25, 0.5 and 1M GuHCl respectively, for 30 min. (*b*) Lanes 1-4, purified RML prions incubated at 25, 50, 75 and 100 °C respectively, for 30 min. (*c*) Purified RML prions incubated at 25 °C (lanes 1 and 2) or 100 °C (lanes 3 and 4) for 30 min and analysed before (-) or after (+) digestion with proteinase K (PK) (10 µg ml^{-1}, 1 h, 37 °C). (*d*) 10% (w/v) RML brain homogenate incubated at 25 °C (lanes 1 and 2) or 100 °C (lanes 3 and 4) for 30 min and analysed before (-) or after (+) digestion with PK (50 µg ml^{-1}, 1 h, 37 °C). Immunoblots were probed with anti-PrP monoclonal antibody ICSM 35.

Supplementary Figure S2

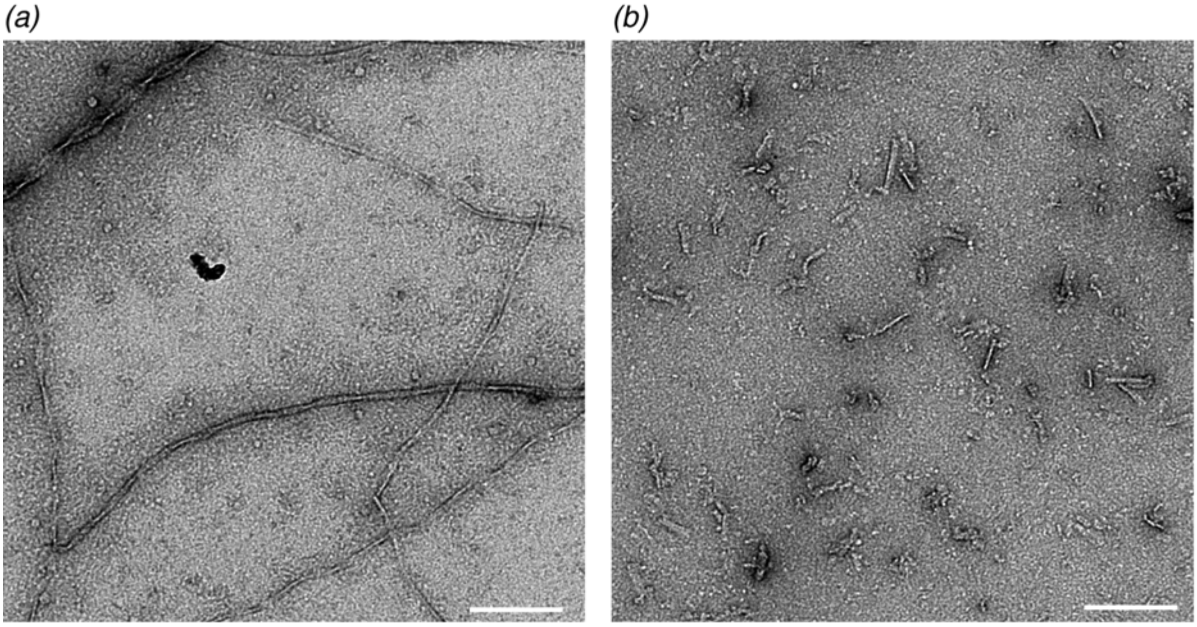

Supplementary Figure S2. Negative stain EM images of non-infectious recombinant PrP fibrils. (*a*) Untreated recombinant PrP fibrils, showing lengths of up to 5 µM. (*b*) Recombinant PrP fibrils sonicated for 10 min. After 10 min sonication all recombinant PrP fibrils are shorter in length (less than 200 nm) with an unaltered width (10-11 nm). Scale bars 200 nm.

Supplementary Table S1. Purified RML prions adhere tightly to electron microscopy grids and are not removed by washing with water

Sample Assayed	% Infectivity applied to grid[a] Experiment 1	% Infectivity applied to grid[a] Experiment 2	% Infectivity applied to grid[a] Experiment 3
Water wash 1	<1	3.2	2.8
Water wash 2	<1	3.0	<1
Total infectivity removed in washes	<1	6.2	<4

[a] Purified RML prions equivalent to ~26,000 intracerebral mouse LD_{50} units (in 2 µl buffer) were applied onto 3 separate glow-discharged 300 mesh carbon coated gold grids and left to dry completely. The grids were then drawn successively through two 20 µl drops of water. Aliquots were withdrawn from each of the water drops and diluted into OptiMEM tissue culture media and tested for their infectivity using the Scrapie Cell Assay. The infectivity titre of 2 µl aliquots of the purified RML prion sample used to load the grids was concomitantly determined enabling the infectivity in the water washes to be expressed as a percentage of that applied to the grids.

Supplementary Table S2. Tissue culture measurement of RML prion infectivity in solution or when bound to electron microscopy grids

Experiment number	LD_{50} units ml^{-1} standard SCA[a]	LD_{50} units ml^{-1} (solution applied to cells)[b]	LD_{50} units ml^{-1} (EM grids applied to cells)[c]
1	1.6×10^7	2.7×10^7	5.6×10^7
2	2.6×10^7	1.4×10^7	5.8×10^7
3	2.6×10^7	3.3×10^7	2.9×10^7
Mean	2.2×10^7	2.4×10^7	4.8×10^7
SEM	0.3×10^7	0.6×10^7	1.0×10^7

[a] 3 µl replicate aliquots of purified RML prions were diluted into tissue culture media and infectivity measured using the standard Scrapie Cell Assay (SCA) format.
[b] 3 µl replicate aliquots of purified RML prions were applied directly into the wells of tissue culture dishes containing 100,000 cells. Aliquots of 4,000 cells were subsequently measured using the SCA.
[c] 3 µl replicate aliquots of purified RML prions were dried onto carbon coated gold electron microscopy grids and placed into wells of tissue culture dishes containing 100,000 cells following the Scrapie Cell Grid Assay (SCGA) format. Aliquots of 4,000 cells were subsequently measured using the SCA.

Supplementary Table S3. Dimensions of purified infectious *ex vivo* PrP rods determined by negative stain electron tomography

Strain	Mean length (nm ± SD)[a]	Mean width (nm ± SD)[a]	Mean thickness (nm ± SD)[a]	Mean width of repeating units (nm ± SD)[a]
RML[b]	177.4 ± 63.7	21.2 ± 2.2	10.6 ± 0.9	6.5 ± 0.5
ME7[c]	171.2 ± 57.3	23.0 ± 3.4	10.8 ± 0.8	6.5 ± 0.4
RML[d]	134.8 ± 29.6	22.2 ± 3.3	10.8 ± 1.1	6.5 ± 0.7

[a] Measurements obtained from tomograms
[b] Purified RML prions from CD1 mouse brain
[c] Purified ME7 prions from C57Bl/6 mouse brain
[d] Purified RML prions from C57Bl/6 mouse brain

Supplementary Table S4. Recombinant PrP fibrils show no infectivity in cell culture

Sample Assayed in SCA	Infectivity LD_{50} units ml^{-1} Experiment 1	Infectivity LD_{50} units ml^{-1} Experiment 2	Infectivity LD_{50} units ml^{-1} Experiment 3	Mean infectivity LD_{50} units ml^{-1} ± SD
Purified RML prions[a]	1.2 x 10^7	1.5 x 10^7	1.1 x 10^7	1.3 ± 0.2 x 10^7
rPrP fibrils[b,c] (non-sonicated) (length >1 µm)	<3300[d]	<3300[d]	<3300[d]	n/a
rPrP fibrils[b] (sonicated, 5min) (length <200 nm)	<3300[d]	<3300[d]	<3300[d]	n/a
rPrP fibrils[b] (sonicated, 10 min) (length <100 nm)	<3300[d]	<3300[d]	<3300[d]	n/a

[a] PrP concentration in the sample was 2.5 µg ml^{-1} prior to dilution 10^{-3} in the SCA.
[b] Recombinant PrP (rPrP) concentration in the sample was 200 µg ml^{-1} prior to dilution 10^{-3} in the SCA.
[c] Preparations of recombinant PrP of these lengths produced no clinical prion disease or evidence for subclinical prion infection (judged by immunohistochemical analyses of brain) when inoculated intra-cerebrally (30 µl of 1 mg ml^{-1} PrP) into CD1 mice after post inoculation periods of 590 – 721 days.
[d] No infectivity was detected. The detection limit of the assay is 3,300 LD_{50} units ml^{-1} in the 200 µg ml^{-1} recombinant PrP preparation.
n/a, not applicable; no infectivity was detected.

Caption for Supplementary Movie S1. Slices through a negative stain tomogram of ME7 prions. ME7 prions were purified from C57BL/6 mouse brain. At least two prion rods can be seen joined in an aggregate (detergent micelles are observed in the background). Each rod is comprised of a pair of intertwined fibres each with a double helical repeating substructure separated by a gap of 8-10 nm. Extra density containing non-repeating substructure is apparent between the fibres and joins the fibres together along the length of the rod. After passing up and down through the density, the individual fibres are traced as blue tubes. An isosurface view of the two rods is shown in figure 5*e*.
